# Trust in information sources as a moderator of the impact of COVID-19 anxiety and exposure to information on conspiracy thinking and misinformation beliefs: a multilevel study

**DOI:** 10.1186/s40359-023-01425-7

**Published:** 2023-11-07

**Authors:** Mustafa Ali Khalaf, Ahmed Maher Shehata

**Affiliations:** 1 Sultan Qaboos University, Muscat, Oman; 2 Minia University, Minia, Egypt

**Keywords:** Conspiracy thinking, Misinformation beliefs, COVID-19 anxiety, Exposure to information sources, Trust in information sources

## Abstract

This study investigates the intricate relationship between exposure to information sources, trust in these sources, conspiracy and misinformation beliefs, and COVID-19 anxiety among 509 Omani citizens aged 11 to 50, representing 11 governorates. Employing structural equation modeling, we not only examine these associations but also explore how trust and COVID-19 anxiety act as moderating variables in this context. Additionally, we delve into demographic factors such as age group, educational level, gender, and place of residence (governorate) to discern potential variations.

Our findings reveal that trust in health experts is inversely related to belief in conspiracy theories, while trust in health experts negatively correlates with exposure to conspiracy and misinformation. Intriguingly, trust in health experts exhibits divergent effects across governorates: it diminishes conspiracy and misinformation beliefs in some regions but not in others. Exposure to personal contacts and digital media, on the other hand, is associated with heightened beliefs in misinformation and conspiracy theories, respectively, in select governorates. These distinctions may be attributed to proximity to Muscat, the capital city of Oman, where various media outlets and policy-making institutions are situated. Furthermore, lower educational attainment is linked to greater belief in conspiracy and misinformation. Females reported higher levels of conspiracy theory beliefs and COVID-19 anxiety while no significant differences were detected in misinformation beliefs.

This study sheds light on the intricate dynamics of misinformation and conspiracy theories in the context of COVID-19 in Oman, highlighting the pivotal roles of trust and COVID-19 anxiety as moderating factors. These findings offer valuable insights into understanding and addressing the spread of misinformation and conspiracy theories during a public health crisis.

## Introduction

The emergence of infectious diseases has historically caused serious problems for both global stability and public health. In 2002, the world grappled with the severe acute respiratory syndrome (SARS) outbreak, which, by 2003, had spread to 29 countries, resulting in 774 deaths, and affecting over 8,000 individuals [1]. In a striking parallel, the novel coronavirus (COVID-19) surfaced in December 2019 in Wuhan, China, and has since evolved into a global concern of unprecedented proportions.

The World Health Organization observed over 659 million confirmed COVID-19 cases as of January 10, 2023, with more than 6.7 million fatalities and more than 13 million vaccination doses given globally [2]. The World Health Organization classified the pandemic as a global health disaster of substantial concern because of its enormous scope, which caused severe suffering and fatalities across the globe [3].

Beyond the direct impacts of the illness, the COVID-19 pandemic has still sparked widespread fear and hysteria, with far-reaching implications that are frequently unrelated to the virus’s actual medical effects [4, 5]. Addressing the psychological effects of COVID-19 has become a pressing concern in the twenty-first century[6].

Numerous research have examined the COVID-19 pandemic’s psychological effects and found a variety of negative effects on mental health and wellbeing. Sleep issues, increased alertness, feelings of helplessness, alterations in mood, health-related concerns, depression, and irritation are among these effects[7–12]. In addition, the epidemic has sparked an alarming rise in conspiracy views and false information, despite intense efforts by governments and groups to stop the virus’s spread [13].

Conspiracy thinking, a well-documented phenomenon during societal crises and health pandemics, has been shown to fuel reluctance to engage in health-related behaviors and foster misunderstandings about the underlying issues [14, 15]. Throughout the COVID-19 pandemic, subjects such as the virus’s origin, severity, and containment have become focal points for conspiracy theories [16] The study of conspiracy thinking has emerged as a burgeoning field, seeking to uncover the factors that influence individuals’ acceptance of such beliefs [17, 18].

Different studies highlighted that conspiracy beliefs led to anxiety and threat [7, 17, 19]. Douglas, Uscinski [20] found that people who believe in conspiracies tend to be more anxious. Additionally, those who experience high levels of anxiety are more susceptible to such beliefs. Misinformation beliefs during covid-19 brought about feelings of anxiety [21]. Within the perspective of continued influence theories, misinformation not only cause poor judgements and decision-making, but it also has a long-lasting effect on people’s reasoning after correction [22]. In their model of false beliefs drivers, Ecker, Lewandowsky [22] posited that misinformation beliefs might come from cognitive drivers (intuitive thinking, cognitive failure, and illusory truth), and socio-affective drivers (source cues, emotion, world view). Verma et al., (2022) found that anxiety level doubled or tripled among twitter users who shared COVID-19 misinformation compared to other users who refused to share misinformation. As such, conspiracy thinking decreases trust in traditional media and increase reliance on social media which transmit misinformation [23, 24]. In other studies, anxiety was not strongly associated with conspiracy theories and misinformation beliefs [24]. We hypothesize that COVID-19 anxiety and sources of information drive individuals to believe in conspiracy theories and misinformation beliefs.

### Problem

The COVID-19 pandemic has significantly impacted Oman, with 399,154 confirmed cases and 4,628 deaths as of January 10, 2023. While the country has made progress in vaccination efforts, with 63.79% of the population having received at least one dose, misinformation remains a significant issue that has not been adequately addressed. In Western cultures, research has shown the importance of conspiratorial and misinformation beliefs in the proliferation of COVID-19 anxiety and “Coronaphobia,“ [25–32] leading to negative effects such as vaccine hesitancy [33]. However, little to no research has been conducted in the Arab Gulf region, specifically in Oman, on the relationship between misinformation beliefs, conspiracy theories, and COVID-19 anxiety.

To address this gap, this study investigates the relationship between misinformation beliefs, conspiracy theories, and COVID-19 anxiety among the Omani population. Research has shown that people believe conspiracy theories when important psychological needs are unmet, such as the desire to satisfy curiosity, avoid uncertainty, and reduce COVID-19 anxiety. By understanding the factors contributing to the spread of misinformation and conspiratorial beliefs in the Omani context, policymakers and health-care professionals can develop targeted interventions to combat this issue.“

### Literature review

In simple terms, “any activity is conspiratorial if it is undertaken in secret by a group of agents who intend some end” [34., p. 24]. Conspiracy theories explain that some kind of conspiracy thinking interprets the incidence of an event. The concept of conspiracy should satisfy three main conditions to be classified as a conspiracy: (a) the conspirators condition (plotters), (b) the secrecy condition, and (c) the goal condition [34]. Considering these three conditions, we can assume that they are inherent in most conspiracy thoughts that accompanied the outbreak of COVID-19 pandemic.

### Conspiracy theories about COVID-19

Conspiracy theories significantly increased following the outbreak of COVID-19. The main purpose of these theories is to explain important events and situations as the malicious acts of third parties [35]. For example, during the pandemic, many people believed that COVID-19 was manufactured in a Chinese laboratory, while others believed that governments exaggerated the severity of the virus as part of a plot to control citizens [28]. People who believe in conspiracy theories tend to engage in irrational behaviors, leading to severe or negative impacts on their health [20]. Additionally, conspiracy theories can harm trust in health institutions and hinder efforts to combat the virus [36].

Scholars have begun to explore the impact of conspiracy theories on individual and public health during the pandemic [25, 37, 38]. For example, Romer and Jamieson [39] examined the impact of conspiracy theories on protective measures in the United States. The results showed that these theories lead to resistance to precautionary actions and vaccination. People avoided wearing masks and getting vaccinated because they believed the virus did not exist and that the vaccine was a means of control. Previous studies have also confirmed the link between conspiracy theories and individuals’ hesitancy to make informed decisions [40, 41].

Uscinski, Enders [32] found that, while many people recognized the seriousness of COVID-19, a significant number of study participants agreed that the danger posed by the virus had been exaggerated and that the virus was purposely produced and spread by other parties. The study highlighted that individuals’ beliefs in conspiracy theories may be driven by denialism, conspiracy thinking, and biased or ideological motivations.

It is difficult to identify a single mechanism that controls the association between education and conspiracy thinking as different psychological mechanisms underlie this relationship e.g., [17]. Not surprisingly, low-educated individuals and those dissatisfied with government actions in response to the pandemic are particularly susceptible to conspiracy theories [42]. Conversely, past research has shown that high levels of education predict low belief in conspiracy theories due to education’s cognitive, emotional, and social outcomes e.g., [17, 43, 44].

It is widely acknowledged in the literature that false beliefs led to an underestimation of the threat and hindered individuals’ efforts to get vaccinated [45]. Different conspiracy theories spread during the pandemic, for example, the belief that 5G technology was the direct cause of the outbreak of COVID-19 [46] and that the virus was manufactured in a Chinese laboratory [35]. As a result, conspiracy theories describing COVID-19 as a hoax were positively associated with less preparedness in containment procedures [30] and predicted vaccine hesitancy [47].

### Misinformation beliefs about COVID-19

The spread of the virus worldwide was accompanied by thousands of people, backed by pseudoscientific treatment ideas and conspiracies [31, 48]. Misinformation has increased on social media due to people believing in this type of information and conspiracy theories, which made social media outlets fertile to germinate a massive amount of misinformation [49, 50].

In the last phases of the pandemic, most false claims were about the vaccine, specifically its side-effect, which cause fear and panic [51, 52]. Some examples of these claims are:

- Vaccines are not safe and cause health risks,
- A way to reduce the population will alter human DNA and causes infertility or death.

In Oman, many refused to get the vaccine because of such rumors [53, 54]. Smith, Ng [55] argued that false information about a cure for the Coronavirus had caused widespread fear and distrust among the public. Vaccine hesitancy was added to the World Health Organization’s top ten global health issues for 2019, according to the WHO’s website. This highlights the concern over the growing trend of individuals refusing vaccines for themselves or their children. The inclusion of vaccine hesitancy on this list predates the COVID-19 pandemic [56]. The authors revealed that misinformation and unfavorable attitudes are very contagious and can lower vaccination rates. For example, during the 2009 swine flu outbreak, the spread of skepticism and unproven hypotheses about vaccine safety affected people’s willingness to be vaccinated. Several studies have found that exposure to anti-vaccination beliefs and misinformation on Twitter has increased vaccine reluctance and refusal and a drop in vaccination uptake [57–59]. Omani citizens and Omani government made positive efforts to deal with misinformation [21].

Health institutions and information systems were not well-prepared to respond to the outflow of infected cases. Although trust in health systems helped shape the public response to the COVID-19 pandemic [60], People’s belief in misleading thoughts decreased their confidence in medical procedures [36]. In his warning in 2020, the Director General of WHO declared the fighting against “infodemic” which spreads faster and more easily than the virus [30]. Misleading information about COVID-19 negatively affected governmental efforts in the struggle with the epidemic leading to unintended deaths [61], and vice versa, the awareness of preventive measures might weaken misinformation beliefs also studies found that there was a strong association between trust in science and belief in misinformation e.g., trusting science more are less likely to believe misinformation [62] Those authors later found that the same trust in science scale was associated with COVID-19 vaccination intention [63]. No doubt that misleading information has a negative impact on taking preventative measures [28]. Conversely, Alper, Bayrak [64] found no association between misinformation beliefs and preventative measures. According to Jovančević and Milićević [65], optimistic people have more preventative measures and less fear than pessimistic people. Kim and Kim [66] claimed that people who perceive danger accept fake news. In this context, McCaffery, Dodd [67] concluded that health knowledge and ways of thinking might have reduced efforts against the pandemic spread in Australia.

### COVID-19 anxiety

The amount of misinformation spread through social networks affected the response to the pandemic, as it had health, psychological and social effects on individuals [25]. The inability to distinguish between facts and misinformation might lead to psychological, health and social distress and may even extend to the economic and political aspects [22, 68]. Many researchers claimed that misinformation increased the level of COVID-19 anxiety among individuals leading to unpredicted actions to avoid infection with covid-19. In addition, it was found that exposure to misinformation increased the level of depression [69]. In order to alleviate these feelings, individuals looked for more information that might comfort them [70].

The COVID-19 outbreak has proved that responding to misinformation is challenging for many reasons. Social, emotional and cultural factors affect the absorption of misinformation, hindering efforts to stop the negative impact of this type of information [71]. In terms of health, Joseph et al. (2022) indicated that spreading false information about the virus through social networks leads to negative results, including reluctance to follow recommendations related to the virus to preserve public health and increasing levels of COVID-19 anxiety and fear. The accumulation of this unfounded information leads to abstaining from vaccinations, which seriously affects public health [72]. Misinformation regarding prevention and treatment measures is particularly harmful because it may directly cause deaths [73]. Misinformation also causes uninformed and rushed health decisions [74].

Psychologically, increased exposure to social media information can negatively affect the community mental health (Hammad and Alqarni [69]. Similarly, in the Arab region, it was believed that the outbreak of the virus has led to continued doubt and uncertainty about the nature of the virus [75]. It has also been proven that the spread of misinformation raises concern and suspicion among the public from the advice given by public health officials [76]. Shehata and Eldakar [75] found that misinformation affects individuals’ health decisions and mental health, leading to increased fear and anxiety. On the other hand, people’s disagreement about the reality of the virus and their exchange of information led to personal and family conflicts [77]. It can also adversely affect health-care infrastructure and society [76].

### Sources of information about COVID-19

People should obtain adequate and accurate information about COVID-19 and vaccine from a trusted source. Public trust building should be a priority through collaboration between citizens and civic institutions in supporting health-care providers [78]. Although “*Exposure to traditional media regularly undertake efforts to debunk conspiracy theories and misinformation"*[24], spread of misinformation about COVID-19 progressed at unprecedented speed worldwide via social media networking sites [79]. Likewise, social media are considered as the main reason behind conspiracy theories as well Georgiou, Delfabbro [42]. Narratives of conspiracy theories and misinformation beliefs were strongly associated with exposure to digital media causing higher feelings of depression [24]. Since COVID-19 preventive precautions were negatively associated with conspiratorial and misinformation beliefs, researchers ought to investigate the psychological, political, and health factors underlying those fake thoughts [80].

A significant number of studies have explored the sources of information adopted by individuals to obtain information about COVID-19 [81–83]. These studies have found a variance in the sources utilized by individuals to seek information related to the virus. Interestingly, it was found that social media outlets represented a significant source of information as many used them for health information. However, using social media outlets produced many problems, as much of the information shared through them is false or misleading [84–86].

Li, Pastukhova [87] and Andika, Kao [88] explored the use of YouTube as a source of information during the COVID-19 pandemic. Both studies revealed that many videos watched by a huge number of viewers contained misleading information that could negatively affect individuals exposed to this information. The studies recommended that health authorities need to collaborate with Youtubers in producing videos that contain reliable health information as the reach of these videos is higher than traditional communication channels.

A study by Mansour, Shehata [89] explored the sources of information utilized by Egyptian physicians working in isolation hospitals. Results indicated that participants prefer to use traditional information sources when dealing with COVID-19 cases, such as research papers and trusted medical databases, with a little emphasis on non-traditional sources, such as social media. Similarly, Tran, Dang [90] focused on Vietnam’s health and community workers. The results outlined that the Internet, online newspapers, and social networks were the most popular channels used by health workers in Vietnam, revealing a lack of proper information literacy practices and a need for tailored programs for information literacy skills. In Taiwan, Wang, Lu [82] found that while many participants, including health-care workers, are using the Internet and social media to obtain health information related to COVID-19, the use of such channels was associated with the participants’ confidence in their ability to obtain reliable information.

Studies also found that individuals utilize other sources of information for health information, including COVID-19 information. A study by Shehata [91] revealed that in addition to social media as a source of health information, personal contacts (family and friends) were among the top sources of information. Other sources, such as authorities’ webpages, newspapers, and magazines, were confirmed to be used by the participants. Notably, many studies confirmed that social outlets such as WhatsApp, Telegram, Instagram, and Facebook were among the highly used sources of health information on the Internet rather than being a source of rumors and misinformation [92–94].

### Trust in sources of information about COVID-19

Social networks have facilitated the dissemination of information worldwide; however, with the infodemic that accompanied the COVID-19 pandemic, individuals could not trust the information they find through social outlets [95]. During the pandemic, COVID-19 misinformation evolved continuously, contributing to the “digital destruction of the mental model” [96]. Therefore, many studies aimed to explore the factors that affect individuals’ trust in the information they read on the Internet. On an individual level, Shehata and Alnadabi [97] investigated the factors that lead undergraduates to trust and share information online using the theory of reasoned action. The results revealed that age, gender, self-efficacy, personal beliefs, and subjective norms play a key role in determining trust in information. Moreover, using digital platforms was associated with lack of basic ethical competencies [98].

Pan, Liu [99] confirmed the previous results, as the study showed that pre-existing beliefs lead to acceptance of misinformation and trust in online information sources. On the other hand, the study claimed that education level and age are not associated with the acceptance of misinformation or trust in online information. Similarly, Shehata [91] explored the health information behavior of undergraduates and revealed that personal beliefs affect individuals’ trust of information, confirming Pan, Liu [99] results. Individuals tend to trust information that is consistent with their beliefs to avoid dissonance in behavior.

Notably, Latkin, Dayton [100] reported a decline in trust in formal information sources in the USA. The study revealed that the state health department and the White House were among the sample’s top untrusted sources of information due to their doubt that politics are playing a part in the spread of COVID-19. Figueiras, Ghorayeb [101] rated health information sources in terms of trust in these sources in UAE. The study argued that trust is influenced by sociodemographic (culture, age, gender) factors. The most trusted sources were physicians, health-care workers, and formal government channels. The results revealed that the use of sources and levels of trust varied based on age, gender, and education. The study also noted that adopting protective behavior affected the level of trust among the sample.

De Coninck, Frissen [24] investigated the relationship between exposure to information sources and conspiracy, and misinformation beliefs; and tested the moderating role of trust in information sources as well as the mediating role of depression and anxiety in eight European, Asian, and American countries during the pandemic. Results indicated that greater exposure to politicians and digital media and personal contacts was associated with higher rate of belief in conspiracy and misinformation, while exposure to traditional media was associated with lower conspiracy and misinformation beliefs. The difference between our study and that of De Coninck et al. is that their study was cross-national comparative research, yet ours is a within-nation comparative study. They used cross-cultural and overseas samples to collect data from USA, UK, New Zealand, Canada, Philippines, Hong Kong, Switzerland, but we recruited only participants from Omani citizens. It is worth noting that we adopted the same instruments.

Overall, studies have shown that the use of information resources varies. The type of information resources used in one region is not necessarily the same in the other as many variables shape the individuals’ behavior and acceptance of information resources. However, it can be said that personal beliefs, self-efficacy, culture, age, gender, and education were the most visible factors in all studies [95, 102, 103].

### Gender differences in conspiracy thinking and misinformation beliefs

Gender has been found to impact the belief in conspiracy theories. Despite the limited research on gender differences in conspiracy thinking, it has been generally observed that men tend to be more inclined to endorse COVID-19 conspiracy theories than females [16] and are more affected by false beliefs [79]. Conversely, Pan, Liu [99] research indicates that females tend to be more accepting of online misinformation than males.

### Age, employment, and level of education

Different studies indicated that people with high level of education are less inclined than those with low level of education to believe in conspiracy theories [17, 43, 44]. To interpret this, Gerosa, Gui [104] argued that people with higher levels of education display higher levels of knowledge. On the other hand, level of education did not have a significant role in believing misinformation. With regard to age, studies in this area are still nascent but some studies, e.g., Douglas, Sutton [105] concluded that young people in middle adolescence are keen on accepting conspiracy theories. Jolley, Douglas [106] believed that conspiracy theories beliefs change across lifespan, and it is not easy to examine conspiracy theories across the lifespan. Concerning employment, countries with high levels of unemployment offer fertile ground for the conspiracy theories [107].

### Study aims

In this study, our primary aims were to elucidate the intricate interplay between conspiracy theories, misinformation beliefs, and COVID-19 anxiety. Specifically, we sought to examine the moderating effect of trust and the mediating effect of COVID-19 anxiety in shaping the relationship between exposure to information sources and individuals’ tendencies towards conspiracy theories and misinformation beliefs. Additionally, we endeavored to explore how these relationships may vary across demographic factors, including age group, educational level, gender, and place of residence (governorate).

### Study hypotheses

In line with this literature, we formulated the following hypotheses

H1. Exposure to digital media will be associated with greater conspiracy and misinformation beliefs.

H2. Exposure to traditional media is expected to be associated with lower conspiracy and misinformation beliefs.

H3. The impact of COVID-19 anxiety and exposure to information sources on conspiracy theories and misinformation beliefs is moderated by trust in these sources.

H4. COVID-19 anxiety is positively associated with conspiracy and misinformation beliefs.

H5. The rate of conspiracy and misinformation beliefs is similar across all governorates.

H6. Conspiracy theories, misinformation beliefs, and COVID-19 would differ significantly according to gender, education level, employment, place of residence, and age.

## Method

### Participants

In this study, a sample of 509 Omani citizens aged between 11 and 50 from 11 governorates in the Sultanate of Oman were recruited to participate. Participants were informed about the study’s aims and objectives and provided information about the data collection process. Demographic information such as age, sex, governorates, education level, and employment was collected to ensure the sample’s representativeness. Participants were administered five online questionnaires measuring conspiracy theories, misinformation beliefs, COVID-19 anxiety, sources of information, and trust in sources of information. The sample consisted of 41% males and 59% females. A detailed description of the demographic characteristics of the sample is provided in Table 1.

**Table 1:** Descriptive results of individual-level variables (in mean or %)

	Muscat	Dhofar	Musandam	Alburaymi	Aldakhiliyah	North Batinah	South Batinah	South Sharqiyah	North Sharqiyah	Aldhahirah	Alwusta	Total
**Age** (mean, SD)	39.5 (6.7)	35.8 (8.3)	36.8 (11.1)	38.7 (8.3)	36.8 (9.6)	36.4 (8.9)	37.3 (9.6)	36.0 (6.7)	38.7 (8.5)	37.5 (11.3)	32.5 (11.3)	37.4 (8.8)
**Job** (*N*)												
Working	67 (79%)	29 (93.5%)	8 (66.6%)	14 (74%)	24 (77%)	67 (65%)	65 (63%)	24 (75%)	35 (66%)	23 (77%)	7 (70%)	363 (71%)
Not working	18 (21%)	2 (6.5%)	4 (33.4)	5 (26%)	7 (23%)	36 (35%)	38 (37%)	8 (25%)	18 (34%)	7 (23%)	3 (30%)	146 (29%)
**Gender** (*N*)												
Male	41 (48%)	6 (19.4%)	8 (66.6%)	7 (37%)	16 (52%)	48 (47%)	28 (27%)	16 (50%)	13 (24.5%)	19 (63%)	5 (50%)	207 (41%)
Female	44 (52%)	25 (80.6%)	4 (33.4)	12 (63%)	15 (48%)	55 (53%)	75 (73%)	16 (50%)	40 (75.5%)	11 (37%)	5 (50%)	302 (59%)
**Educational Level** (*N*)												
High School	14 (16.5%)	5 (16%)	3 (25%)	2 (11%)	6 (19%)	25 (24%)	38 (37%)	3 (9%)	12 (22%)	5 (17%)	4 (40%)	117 (23%)
Bachelor	51(60%)	20 (54.6%)	7 (58%)	12 (63%)	21 (68%)	63 (61%)	56 (54.3%)	24 (75%)	36 (68%)	20 (67%)	5 (50%0	315 (62%)
Master or PhD	20 (23.5)	6 (19.4)	2 (17%)	5 (26%)	4 (13%)	15 (15%)	9 (8.7%)	5 (16%)	5 (10%)	5 (16%)	1 (10%)	77 (15%)
**Conspiracy (**mean)	39.9	36.2	40.7	38.5	39.8	39.7	40.3	38.7	41.1	40.3	39.4	39.6
**Misinformation**	21.5	24.0	30.0	23.9	21.1	21.3	23.0	26.5	18.9	19.6	27.1	22.2
**COVID-19 anxiety**	11.8	11.7	12.4	10.6	12.2	11.4	12.7	11.1	10.9	10.8	11.4	11.7
**Exposure to info. sources**												
Health experts	7.8	7.6	8.6	7.5	8.3	7.9	8.1	7.9	7.9	8.1	7.7	7.9
Digital media	10.7	9.4	11.1	10.8	10.3	10.0	11.2	10.9	9.1	9.8	9.5	10.3
Traditional media	10.3	9.7	12.3	10.2	10.4	10.1	10.3	10.8	8.5	9.2	8.9	10.0
Personal contacts	2.7	2.8	2.8	2.4	2.7	2.8	2.8	2.8	2.6	2.6	2.6	2.7
**Trust in info. Sources**												
Health experts	21.8	19.9	23.3	21.4	21.5	20.5	21.3	22.3	18.8	20.1	17.6	20.9
Digital Media	4.7	4.2	6.1	4.6	4.9	4.6	4.6	5.1	3.7	4.3	4.6	4.6
Traditional Media	5.6	5.0	5.7	5.6	5.2	5.3	5.8	5.3	4.8	4.7	5.3	5.4
Personal contacts	4.4	3.9	5.1	4.3	5.3	4.6	4.8	5.0	4.1	4.7	3.2	4.6
*N*	85	31	12	19	31	103	103	32	53	30	10	509

### Sampling

The researchers utilized a non-probability convenience sampling technique to select the participants for the study. The participants were selected through WhatsApp groups in Oman. Ten groups were selected to ensure representation from all Oman governments. The questionnaire was distributed through these groups from September 2022 to November 2022.

### Measures

#### COVID-19 brief anxiety scale (CO-BAS-4)

Numerous studies have indicated that measurement tools used in research should possess robust psychometric properties [108, 109]. In line with these findings, CO-BAS was selected for assessing COVID-19-related anxiety in the present study, as it has been shown to possess valid and reliable psychometric properties within an Omani context. The scale was composed of four items measuring four facets of disease anxiety and was developed and validated using a large sample of participants from different age groups. The responses ranged between 1 strongly disagree to 5 strongly agree. The minimum score is 5 and the maximum score is 25. Results from the Exploratory and confirmatory factor analyses supported a unidimensional structure, with the factorial structure of the scale explaining 58.74% of the cumulative variance. Furthermore, confirmatory factor analysis (CFA) revealed that the scale’s goodness-of-fit indices fell within the acceptable range. Criterion-related validity was established through the scale’s association with the COVID-19 Anxiety Scale (r = .760, p < .01) [6] and the Work and Social Adjustment Scale (r = .502, p < .01) [110]. The scale’s internal consistency reliability was ensured using both Cronbach’s alpha and McDonald’s omega, with both measures yielding values of 0.810, indicating adequate internal consistency reliability [111, 112].

#### Conspiracy theories and misinformation beliefs questionnaires

In the present study, we employed questionnaires developed by De Coninck, Frissen [24] to measure participants’ beliefs in conspiracy theories and misinformation related to COVID-19. The conspiracy theories questionnaire consisted of 6 items, each with ten response options ranging from 1 (fully disagree) to 10 (fully agree). The minimum score is 6 and the maximum score is 60. An example item from the questionnaire is “I believe that coronavirus was made intentionally in a laboratory.“ The questionnaire showed adequate internal consistency reliability, as evidenced by a Cronbach’s Alpha coefficient of 0.86. Exploratory factor analysis supported a one-factor solution for the questionnaire. In our study, we also computed and reported the internal consistency reliability of the questionnaire using both Cronbach’s Alpha (α = 0.619, SE = 0.03, 95% lower CI = 0.557, upper CI = 0.673) and McDonald’s omega (ω = 0.910, SE = 0.05, 95% lower CI = 0.795, upper CI = 0.988). The measurement of misinformation beliefs was conducted using a separate questionnaire consisting of 5 items, with examples such as “I believe that spraying the alcohol or chlorine all over my body will kill the new coronavirus.“ We computed the internal consistency reliability of this questionnaire using both Cronbach’s Alpha (α = 0.666, SE = 0.03, 95% lower CI = 0.608, upper CI = 0.713) and McDonald’s omega (ω = 0.683, SE = 0.03, 95% lower CI = 0.627, upper CI = 0.726) and found them to be adequate.

#### Exposure to information sources questionnaire

Eight items were used to measure four main sources of information citizens use to get news about COVID-19. The four dimensions are public health experts, traditional media (TV, newspapers, radio), digital media (Facebook, YouTube what’s app, Twitter, or telegram, etc.), and personal contacts (family and friends). Response categories ranged from 1 never to 4 mainly/ always. The minimum score is 8 and the maximum score is 32. De Coninck, Frissen [24] verified its factorial validity and extracted the previous four components, and computed Cronbach’s Alpha, whose values ranged between 0.67 and 0.73. In our study, Cronbach’s Alpha and McDonald’s omega were computed for questionnaire of exposure to information sources and reported respectively (α = 0.741, SE = 0.02, 95% lower CI = 0.699, upper CI = 0.780), (ω = 0.731, SE = 0.02, 95% lower CI = 0.672, upper CI = 0.771).

#### Trust in information sources questionnaire

In the present study, we employed a seven-item measure of trust in information sources, as utilized by De Coninck, Frissen [24], to assess participants’ trust in four sources of information related to COVID-19. The response options ranged from 1 (do not trust at all) to 10 (fully trust). The minimum score is 7 and the maximum score is 70. The psychometric properties of this measure were evaluated using both Cronbach’s alpha and McDonald’s omega. The results revealed adequate internal consistency reliability, with a Cronbach’s alpha of 0.896 (SE = 0.01, 95% CI = 0.878 − 0.911) and a McDonald’s omega of 0.899 (SE = 0.01, 95% CI = 0.880 − 0.914).

### Statistical analysis

We used SPSS 26.0 and AMOS 22.0 for data analysis. Descriptive statistics were calculated for all study variables (Table 1). Associations between conspiratorial, misinformation beliefs, COVID-19 anxiety, exposure to information and trust in information sources were examined using Pearson correlation coefficient (Table 2). According to De Coninck, Frissen [24], standard Error of the Mean (SEM) “can test all mediated effects simultaneously if there are multiple mediators” (p.5). Moreover, it is better than ordinary least squares regression as it considers the measurement errors and provides accurate estimations of effects. Finally, it is a robust statistical technique for a confirmatory approach. Accordingly, we used the structural equation model to assess the association between trust sources, exposure to information sources, COVID-19 anxiety, and conspiracy theories and misinformation beliefs. The moderator is trust, the dependent variables are conspiracy theories and misinformation beliefs, while the independent variables are gender, education level, health experts, digital media, traditional media, personal contacts, and COVID-19 anxiety.

**Table 2:** Pearson correlations, means, and standard deviations of the study variables. shows that conspiracy theories and misinformation beliefs are significantly positively or negatively associated with all exposure to information sources and trust in these sources except exposure to personal contacts and health experts, while COVID-19 was significantly associated with all exposure sources and trust

	M	SD	1	2	3	4	5	6	7	8	9	10
Conspiracy theory	39.6	10.2	1									
Misinformation beliefs	22.23	9.6	0.140**	1								
COVID-19 anxiety	11.7	4.7	0.225**	0.163**	1							
**Exposure**: Health experts	7.9	2.0	− 0.001	0.133**	0.212**	1						
**Exposure**: Digital media	10.3	4.9	− 0.196**	0.208**	0.111**	0.422**	1					
**Exposure**: Traditional media	10.0	4.6	− 0.140**	0.170**	0.140**	0.409**	0.783**	1				
**Exposure**: Personal contacts	2.7	0.94	0.019	0.052	0.096*	0.400**	0.295**	0.287**	1			
**Trust**: Health experts	20.9	9.0	− 0.175**	0.185**	0.105*	0.390**	0.895**	0.871**	0.301**	1		
**Trust**: Digital Media	4.6	2.6	− 0.123**	0.176**	0.174**	0.423**	0.680**	0.895**	0.228**	0.679**	1	
**Trust**: Traditional Media	5.4	2.7	− 0.147**	0.152**	0.140**	0.381**	0.918**	0.731**	0.292**	0.797**	0.615**	1
**Trust**: Personal contacts	4.6	2.6	− 0.075	0.269**	0.195**	0.346**	0.333**	0.454**	0.165**	0.354**	0.488**	0.274**

Note, N = 509, *p < .05; **p < .01

### Two-level SEM analysis

The data was collected through a self-administered online questionnaire. The questionnaire was designed using Google Forms and was distributed through WhatsApp groups. The researchers selected ten groups consisting of participants from all Oman governments, and the questionnaire was distributed from September 2022 to November 2022.

To ensure the ethical process, the researchers obtained approval from the Research Ethics Committee at Sultan Qaboos University. Participants were informed about the purpose of the study, and their participation was voluntary. They were also assured of confidentiality, and their data was kept anonymous. The participants provided their informed consent statement in the introduction of the questionnaire.

The data collection process was designed in a way that the participants’ privacy was protected. The participants were not asked to provide personal information, and the data was stored on the researcher’s computer. The researchers followed the guidelines provided by the Research Ethics Committee to ensure the ethical and safe conduct of the study.

We analyzed the path model on the participants and region levels through Multilevel Structural Equation Modeling (ML-SEM) method. ML-SEM is a method for simultaneously comparing the complicated relationships among the latent variables on different levels. This technique offers advantages of the SEM and multilevel models and is adequately flexible for assessing the fitness of models and computing level-2 outcomes [113]. We considered regions as clusters. Estimations were made using the Maximum likelihood Ratio (MLR) method [114]. Evaluation of the ML-SEM model was based on the following criteria recommended by Mueller and Hancock [115]: the root mean square error of approximation (RMSEA) and the standardized root mean square residual (SRMR) not being larger than 0.05, and the Comparative Fit Index (CFI) and the Tucker–Lewis index (TLI) not being smaller than 0.95.

In this study, we used a two-step process to evaluate the two-level model. The variables’ descriptive statistics were evaluated in the first phase in order to choose the best estimation method. The second stage determines whether the variability between clusters (i.e., the between-level variability) is significant enough to justify an analysis at the cluster level for those variables that exhibit both between-level and within-level variation. Examining the intra-class correlations, which estimate the proportion of between-cluster variance to the overall variance for each variable, will provide this information. There is insufficient between-level variation to support the second level analysis if the proportion is close to zero for the majority of the variables. A typical cut-off point for evaluating this variation is.05 [116].

No variable has an extreme distribution or severe kurtosis, according to the univariate results, indicating that the data are appropriate for ML estimation. Utilizing Mplus 7.4’s Maximum Likelihood Ratio (MLR) approach, we fitted the two-level SEM [117].

The intraclass correlation estimates obtained in the second preliminary step above were as follows: 0.061 (Conspiracy theories) and 0.083 (Misinformation beliefs). These estimates imply that, as would be expected, within-level variability is typically far bigger than between-level variability. They do, however, show that there is sufficient between-cluster variation to move forward with the two-level analysis because they are above the.05 cut-off value e.g., [118], especially if we take consider that these estimates are generally attenuated (biased downward) because of measurement error.

## Results

Table 2 provides a comprehensive presentation of Pearson correlations, means (M), and standard deviations (SD) pertaining to the study’s variables under investigation. Within this tabular representation, each variable is denoted by its corresponding abbreviation, appearing both along the horizontal and vertical axes of the table, thereby affording a structured examination of their interrelationships.

For a more holistic understanding of the dataset, M and SD values for each variable are thoughtfully supplied. Mean values offer insight into the central tendency of each variable, while standard deviations elucidate the extent of variability within the dataset.

Of paramount significance, the correlation coefficients are thoughtfully arrayed within the matrix. These coefficients, assigned numerical designations from 1 to 10 in the table’s header, facilitate a meticulous exploration of associations and relationships between variables.

It is imperative, however, to delineate the critical interpretation of this matrix. The diagonal, traversing from the top-left to the bottom-right corner, elucidates self-correlations for each variable. Intrinsically, these self-correlations invariably yield a value of 1, serving as reference points for gauging the magnitude and direction of relationships with other variables.

Conversely, the cells situated off the diagonal, both below and above it, expose correlations between variable pairs. Positive correlations, identifiable through positive values such as 0.140**, denote a direct proportionality: an increment in one variable corresponds to a concomitant increase in the other. In stark contrast, negative correlations, manifested through negative values like − 0.175**, signify an inverse relationship, suggesting that an elevation in one variable coincides with a reduction in the other.

Table 2 goes beyond these correlations to present the outcomes of the Multilevel Structural Equation Modeling (ML-SEM) model. This model evaluation endeavors to determine the model’s appropriateness in representing the data. The model’s adequacy is established, demonstrating favorable fit statistics (𝝌2 = 286.62, df = 42, p < .01, RMSEA = 0.001, TLI = 0.990, CFI = 0.992, SRMR within = 0.002, SRMR between = 0.028).

Table 3. shows the ML-SEM model results. The ML-SEM model resulted in adequate fit to the data (𝝌2 = 286.62, df = 42, p < .01, RMSEA = 0.001, TLI = 0.990, CFI = 0.992, SRMR_within_ = 0.002, SRMR_between_=0.028). The results from the analysis of the direct of the (Health experts, Digital media, Traditional media, Personal contacts, and COVID-19 anxiety) variables and interaction effects of the trust and (Health experts, Digital media, Traditional media, and Personal contacts) variables in the ML-SEM model are listed in Table 3. On the student level, the Conspiracy theories and Misinformation beliefs are caused by different sources. On this level, the Conspiracy theories caused by gender (b=-0.149, P < .001) but negative effect of student gender in favor of female students (0 female and 1 male), TR×BC (b = 0.116, P = .001), and COVID-19 anxiety (b = 0.200, P = .001). the Misinformation beliefs caused by TR×BC (b = 0.232, P < .001), and COVID-19 anxiety (b = 0.117, P < .01). On the region level, the Conspiracy theories caused by Health experts (b = 0.685, P < .05). the Misinformation beliefs caused by personal contacts (b=-0.805, P < .001).

**Table 3:** The Effects (standard errors) of variables in ML-SEM model

Variables	Conspiracy theories					Misinformation beliefs				
	Est.	S.E.	Z	P-Value	CI (95%)	Est.	S.E.	Z	P-Value	CI (95%)
Within Level										
Education	0.008	0.039	0.217	0.828	− 0.057;0.073	-0.041	0.028	-1.433	0.152	− 0.090;0.008
Gender	-0.149	0.038	-3.895	0.000***	− 0.214; − 0.084	0.040	0.057	0.701	0.483	− 0.055;0.135
HE	0.024	0.059	0.408	0.684	− 0.069;0.117	-0.008	0.060	-0.131	0.896	− 0.103;0.087
DM	-0.291	0.175	-1.669	0.095	− 0.520; − 0.062	0.342	0.221	1.548	0.122	0.064;0.620
TM	0.176	0.188	0.938	0.348	− 0.083;0.435	-0.227	0.309	-0.734	0.463	− 0.626;0.172
PC	0.071	0.037	1.935	0.053	0.011;0.131	-0.026	0.040	-0.667	0.505	− 0.093;0.041
TR×HE	-0.100	0.165	-0.607	0.544	− 0.320;0.120	0.088	0.279	0.317	0.751	− 0.287;0.463
TR×DM	-0.201	0.153	-1.313	0.189	− 0.418;0.016	0.051	0.194	0.264	0.792	− 0.194;0.296
TR×TM	0.126	0.122	1.029	0.304	− 0.052;0.304	-0.178	0.146	-1.218	0.223	− 0.384;0.028
TR×PC	0.116	0.036	3.200	0.001***	0.056;0.176	0.232	0.044	5.226	0.000***	0.159;0.305
Anxiety	0.200	0.061	3.269	0.001***	0.099;0.301	0.117	0.044	2.684	0.007**	0.045;0.189
Between Level										
HE	0.685	0.340	2.015	0.044*	0.019;1.351	0.133	1.123	0.118	0.906	-1.750;2.016
DM	0.646	0.753	0.857	0.391	− 0.579;1.871	-0.795	1.006	-0.790	0.429	-1.793;0.203
TM	-0.715	1.045	-0.685	0.493	-2.152;0.722	1.416	1.451	0.976	0.329	0.186;2.646
PC	0.046	0.544	0.084	0.933	-1.358;1.450	-0.805	0.242	-3.326	0.000***	-1.279; − 0.331
TR×HE	0.347	0.759	0.457	0.648	− 0.719;1.413	-0.577	0.908	-0.635	0.525	-2.075;0.921
TR×DM	0.345	1.482	0.233	0.816	− 0.826;1.516	-0.455	1.960	-0.232	0.816	-1.787;0.877
TR×TM	0.443	0.635	0.697	0.486	− 0.231;1.117	-0.677	0.860	-0.787	0.431	-1.446;0.092
TR×PC	0.614	1.085	0.566	0.572	− 0.431;1.659	-0.721	1.727	-0.417	0.676	-2.450;1.008
Anxiety	-0.189	1.355	-0.140	0.889	-1.035;0.657	0.269	2.164	0.124	0.901	-1.040;1.578

Note, HE = Health experts, DM = Digital media, TM = Traditional media, PC = Personal contacts, CI = Confidence Intervals

### Findings of H1, H2, and H3

**H1.** Exposure to digital media will be associated with greater conspiracy and misinformation beliefs.

**H2.** Exposure to traditional media is expected to be associated with lower conspiracy and misinformation beliefs.

**H3.** The impact of COVID-19 anxiety and exposure to information sources on conspiracy theories and misinformation beliefs is moderated by trust in these sources.

### Multilevel SEM findings

Findings of within groups analysis revealed that gender has a positive effect on conspiracy theories with males scoring higher than females. Likewise, COVID-19 anxiety has a positive effect on conspiracy theories and misinformation beliefs. This implies that as COVID-19 anxiety increases, individuals became more likely to believe in conspiracy theories and misinformation beliefs. Trust has a positive moderating effect on the relationship between personal contacts and conspiracy theories. Similarly, trust has a positive moderating effect on the relationship between personal contacts and misinformation beliefs. Findings of between groups analysis indicated that health experts as a source of information and COVID-19 anxiety have positive impacts on conspiracy theories. Personal contacts as a source of information has a negative effect on misinformation beliefs.

The results revealed that trust in health experts in the Musandam and South Batinah governorates was positively associated with a decrease in conspiracy and misinformation beliefs, consistent with previous research. In contrast, in the North Batinah governorate, high exposure to personal contacts was positively associated with stronger belief in misinformation. In the Aldakhiliyah governorate, greater exposure to digital media was associated with increased belief in conspiracy theories. Furthermore, in the South Sharqiyah governorate, exposure to digital media was positively associated with a high rate of misinformation beliefs, potentially due to a lack of trust in health experts appearing on digital media. Additionally, it is worth noting that about 20% of the sample were low educated (holders of high school certificates), which may also contribute to the high rate of conspiratorial thoughts and misinformation beliefs.

Exposure to sources of information and its interaction with trust in these sources were found to be significant in only two governorates, Musandam and Aldakhiliyah. Specifically, in Musandam, exposure to health experts and trust in health experts were associated with lower belief in conspiracy theory, while exposure to digital and traditional media was associated with higher belief in misinformation. These findings support H1 and reject H2. In this context, Hollander [119] suggested that traditional media exerts efforts to decrease conspiracy and misinformation beliefs. In contrast, in the Aldakhiliyah governorate, Low misinformation beliefs were connected with the interaction of exposure to and trust in conventional media, digital media, and personal relationships. Additionally, less conspiracy theories were found in Aldakhiliyah when people were exposed to digital media. With the exception of Dhofar and Alburaymi, all governorates showed a negligible correlation between exposure to and interactions with conspiracy and false information. Increased conspiracy views in Musandam and larger disinformation beliefs in Muscat were both correlated with trust in personal interactions. In the Dhofar Governorate, it was also discovered that confidence in digital media moderated the association between exposure to digital media and misleading beliefs. This finding partially supported hypothesis 3.

### H4. COVID-19 anxiety is positively associated with conspiracy and misinformation beliefs

Regarding COVID-19 anxiety (see Table 2), it was positively associated with conspiracy in three governorates: Muscat, North Batinah, and South Batinah. Similarly, it was positively associated with misinformation in three governorates: North Batinah, South Sharqiyah, and Aldhahirah, while negatively correlated to conspiracy theories in Musandam. Thus, we can confirm H4.

### H5. The rate of conspiracy and misinformation beliefs is similar across all governorates

One-way ANOVA was conducted to compare the effect of the governorate of residence on conspiracy theory, misinformation beliefs, and COVID-19 anxiety. Comparisons were made between eleven governorates. There was no significant difference in Conspiracy theory and COVID-19 anxiety among them, so we did not perform post hoc test for both variables, while there were significant differences among the eleven governorates in misinformation beliefs at the p < .001 level for the three levels F (10.498) = 3.06, p = .001. LSD post hoc test results revealed that citizens of Musandam (M = 30) were significantly higher in misinformation beliefs compared to all other governorates, namely, Alwusta (M = 27.1), South Sharqiyah (M = 26.5), Dhofar (M = 24.0), Alburaymi (M = 23.9), South Batinah (M = 23.0), Muscat (M = 21.5), North Batinah (M = 21.3), Aldakhiliah (M = 21.1), Aldhahirah (M = 19.6), and the lowest rate of misinformation beliefs was found among citizens of North Sharqiyah governorate (M = 18.9). Taken together, these differences support H5.

### H6. Conspiracy theories, misinformation beliefs, and COVID-19 would differ significantly according to gender, education level, employment, place of residence, and age

The present study’s findings regarding demographic factors, specifically the governorate of residence, were mixed. One-way ANOVA revealed significant differences among governorates in terms of conspiracy theory beliefs (as seen in Table 4), while no significant differences were found in COVID-19 anxiety among governorates. Specifically, citizens from the North Sharqiyah (M = 41.1), Musandam (M = 40.7), and South Batinah (M = 40.3) governorates reported the highest mean scores for conspiracy theory beliefs, while respondents from the South Sharqiyah (M = 38.7), Alburaymi (M = 38.5), and Dhofar (M = 36.2) governorates reported the lowest mean scores. Similarly, significant differences were found among governorates in terms of misinformation beliefs, with citizens from the Musandam (M = 30.0), Alwusta (M = 27.1), and South Sharqiyah (M = 26.5) governorates reporting the highest levels of misinformation beliefs, while respondents from the Aldakhiliyah (M = 21.1), Aldhahirah (M = 19.6), and North Sharqiyah (M = 18.9) governorates reported the lowest levels of misinformation beliefs. These findings may be explained by the proximity to the capital city of Oman, Muscat, where various media sources and policy-making institutions are located. Additionally, this finding is consistent with previous research that has found that low education is associated with high belief in conspiracy and misinformation [42], and vice versa, high educational level predicts low belief in conspiratorial thoughts [17, 43, 44]. These findings are partially consistent with previous literature [24] and partially support H6.

**Table 4:** One-way ANOVA for governorate of residence on conspiracy, misinformation, and anxiety

Variables	Source of variance		Sum of squares	DF	Mean square	F	*P*
Conspiracy theory	Between Groups		637.13	10	63.71	0.611	0.805
	Within Groups		51961.78	498	104.34		
	Total		52598.91	508			
Misinformation beliefs	Between Groups		2714.26	10	271.43	3.063	0.001
	Within Groups		44132.76	498	88.62		
	Total		46847.02	508			
COVID-19 anxiety	Between Groups		217.012	10	21.70	0.994	0.447
	Within Groups		10872.15	498	21.83		
	Total		11089.16	508			

### Education levels differences

One-way ANOVA was utilized to evaluate the effect of education levels on conspiracy theories, misinformation beliefs, and COVID-19 anxiety. The education levels compared were high school, Bachelor, and Master/PhD holders (as seen in Table 5). No significant difference in conspiracy theories and misinformation beliefs was found among the educational group levels, so post-hoc tests were not performed for these variables. In contrast, there was a significant difference in COVID-19 anxiety among the three levels of education at the p < .001 level, with F (2.506) = 9.26, p = .000. The LSD post-hoc test revealed that high school holders had significantly higher COVID-19 anxiety (M = 13.2, SD = 4.9) compared to Bachelor holders (M = 11.3, SD = 4.6) and Master/PhD holders (M = 10.7, SD = 4.3). Additionally, Bachelor holders were found to have higher COVID-19 anxiety compared to Master/PhD holders. This finding partially confirms H6.

**Table 5:** One-way ANOVA for educational level on conspiracy, misinformation, and anxiety

Variables	Source of variance	Sum of squares	DF	Mean square	F	*P*
Conspiracy theories	Between Groups	98.453	2	49.23	0.474	0.623
	Within Groups	52500.46	506	103.76		
	Total	52598.91	508			
Misinformation beliefs	Between Groups	253.03	2	126.51	1.374	0.254
	Within Groups	46593.99	506	92.08		
	Total	46847.02	508			
COVID-19 anxiety	Between Groups	391.61	2	195.80	9.262	0.000
	Within Groups	10697.55	506	21.14		
	Total	11089.16	508			

### Age group differences

One-way ANOVA was used to evaluate the effect of age groups on conspiracy theories, misinformation beliefs, and COVID-19 anxiety. The age groups compared were (11–34 years), (35–41 years), and (42–58 years) (as seen in Table 6). A significant difference in conspiracy theories and misinformation beliefs was detected among the age groups at the p < .001 level, with F (2.506) = 8.34, p = .000 and F (2.506) = 16.97, p = .000, respectively. However, no significant difference was found in COVID-19 anxiety among the age groups. The results of the LSD post-hoc test revealed that the (35–41 year) age group had significantly higher conspiracy theories (M = 41.0, SD = 9.7) compared to the (11–34 years) age group (M = 37.1, SD = 9.9) and the (42–50 years) age group (M = 40.8, SD = 10.4). Conversely, the (11–34 years) age group had significantly higher misinformation beliefs (M = 25.6, SD = 8.9) than the (35–41 year) age group (M = 20.7, SD = 9.7) and the (42–50 years) age group (M = 20.3, SD = 9.4). This result partially supports H6. No significant difference in COVID-19 anxiety was detected between the three age groups.

**Table 6:** One-way ANOVA for age groups on conspiracy, misinformation, and anxiety

Variables	Source of variance		Sum of squares	DF	Mean square	F	*P*
Conspiracy theories	Between Groups		1679.40	2	839.701	8.34	0.000
	Within Groups		50919.51	506	100.631		
	Total		52598.91	508			
Misinformation beliefs	Between Groups		2944.57	2	1472.282	16.97	0.000
	Within Groups		43902.45	506	86.764		
	Total		46847.02	508			
COVID-19 anxiety	Between Groups		57.04	2	28.519	1.31	0.271
	Within Groups		11032.12	506	21.803		
	Total		11089.16	508			

### Gender and employment differences

A significant gender difference was found in terms of conspiracy theory beliefs, with females reporting higher levels of conspiracy theory beliefs (see Table 7). Conversely, males reported similar levels of misinformation beliefs. In terms of COVID-19 anxiety, females reported higher levels of anxiety. Additionally, differences were detected in terms of employment status, with employed individuals showing a higher tendency to adopt conspiracy theories, while unemployed individuals reported higher levels of misinformation beliefs and COVID-19 anxiety. These findings support H6.

**Table 7:** T-test results for gender and job differences in conspiracy, misinformation, and anxiety

Variables	Group		*N*	*M*	*SD*	*T*	*DF*	*P*	Eta[2]
Conspiracy theory	M		207	37.3	9.9	-4.176	507	0.000	0.033
	F		302	41.1	10				
Misinformation beliefs	M		207	22.6	8.8	0.744	507	0.457	0.001
	F		302	22.0	10.1				
COVID-19 anxiety	M		207	11.0	4.1	-2.668	507	0.008	0.014
	F		302	12.1	5.0				
Conspiracy theory	EM		363	39.6	9.7	0.240	507	0.811	0.000
	UN		146	39.4	11.4				
Misinformation beliefs	EM		363	22.0	9.4	− 0.980	507	0.328	0.002
	UN		146	22.9	10.1				
COVID-19 anxiety	EM		363	11.5	4.5	-1.22	507	0.224	0.003
	UN		146	12.1	5.0				

Note, M = male, F = female, EM = employee, UN = Unemployed

## Discussion

This study, informed by prior research on conspiracy and misinformation beliefs, is one of the first to investigate the effects of exposure and trust in information sources on conspiracy theories, misinformation beliefs, and anxiety in the Sultanate of Oman. The findings indicate a relationship between exposure to sources of information about the pandemic and conspiratorial thoughts and misinformation beliefs in Oman, which is moderated by trust in these sources.

The spread of conspiracy theories and misinformation beliefs in parallel with the COVID-19 pandemic has been well documented in prior research. Systematic reviews have shown that numerous myths and rumors were highly prevalent during the pandemic [25] and represented an ongoing challenge that hindered the fight against the pandemic [120] and weakened the efficiency of health-care institutions. Despite this, research into conspiracy theory and misinformation about COVID-19 is still incipient in the Omani context.

Although people tend to trust traditional media sources, they were found to be more responsive to conspiracy theories and misinformation beliefs during the pandemic [24]. This highlights the importance of understanding how exposure to sources of information and trust in these sources shape beliefs and attitudes in the context of the pandemic.

Our results indicated that exposure to digital and traditional media was associated with low conspiracy and misinformation beliefs. This is perhaps, as Zhong, Luo [121] suggested, because mass media are supposed to be a source of credible information. Conversely, Shehata, Al-Suqri [21] concluded that misinformation had created doubt and anxiety among Omani citizens and hindered many their ability to take countermeasures and obtain reliable data.

De Coninck, Frissen [24] theorize that human dynamics involved in COVID-19 are similar to those involved in spreading misinformation beliefs and conspiratorial thoughts. This may help to explain the coincidence of conspiracy and misinformation with the pandemic. The present study found significant differences in conspiracy theories, misinformation beliefs, and anxiety among governorates, with some governorates being more affected than others. It is possible that the governorates hit hardest by the pandemic had the most significant increase in anxiety prevalence, as Santomauro, Herrera [122] suggested bearing in mind that the latter examined countries and territories not governorates.

Recent studies have also investigated the impact of exposure to social media on mental health during the pandemic. Some studies have found that exposure to social media was associated with increased levels of depression and prior diagnosis of post-traumatic stress disorder during the first months of the pandemic [123]. However, other studies have found that digital media did not significantly increase mental health problems or lead to suicidal ideation, as there are no sufficient evidence-based findings to support these claims [124]. Additionally, some studies have suggested that excessive use of social media might be an adaptive mechanism to reduce the level of distress [125]. These findings are consistent with previous research, which found that digital media was the most frequent source of information for people diagnosed with anxiety [126].

Concerning gender differences in COVID-19 anxiety, this study found insignificant differences between males and females. This may be attributed to the fact that both genders have similar feelings of uncertainty, panic, and fear of infection. Whether male or female, citizens were afraid of the high rate of deaths and the ongoing increase of infected cases. Additionally, the mutations of the virus and the parallel spread of infodemics likely exacerbated their anxiety. It is also worth noting that feelings of doubt and uncertainty are a self-evident truth during global health pandemics such as COVID-19 [24]. Our insignificant gender differences are in line with previous studies, which found that the COVID-19 pandemic has caused a substantial increase in the prevalence of anxiety [126]. However, our results are inconsistent with previous studies, which found that females and younger age groups were affected more by the pandemic than males and older age groups in terms of anxiety [122].

This study examined the difference in COVID-19 anxiety attributable to education level. Results indicated that high anxiety rates were associated with lower education levels. This finding is consistent with previous research conducted by Zhong, Luo [121], which indicated that education level predicts positive changes in attitudes during pandemics. This highlights the importance of educating individuals during health pandemics and thereafter. However, our results are inconsistent with those of Pan, Liu [99], who found no significant association between education level and age with the acceptance of misinformation or trust in online information.

In terms of age differences in conspiracy and misinformation beliefs, our results showed that adults had higher levels of conspiracy and misinformation beliefs compared to younger age groups. This finding is consistent with prior research, which posits that low ability to detect misinformation is associated with greater chronological age. Additionally, different psychological and contextual factors, such as analytical reasoning, affect, and news consumption frequency, determine the extent to which people can detect false news. Despite this, there is limited evidence of age effects on detection of misinformation. However, older individuals tend to consume more fake news than younger age groups [127], which supports our findings.

Significant gender differences in conspiracy and misinformation beliefs were also observed. However, these results were inconsistent with previous research, which found that men had more COVID-19 conspiracy theories than females [16] and were mostly influenced by fake beliefs [79]. Additionally, our findings were inconsistent with the results of Pan, Liu [99], who found that females tended to accept online information more than males. It is possible that uncertain mental conditions, such as anxiety, lead females to trust more misinformation as a form of comfort.

Our results also showed that unemployed citizens had higher levels of misinformation beliefs and COVID-19 anxiety than employees. Factors such as lockdown, social distancing, fear of job loss, investments escape, and reduced employment opportunities may have contributed to the spread of misinformation and anxiety. These findings are consistent with research conducted by Yao and Wu [128] who found that mental disorders are more frequent among unemployed individuals. Furthermore, our results regarding age differences are consistent with the findings of Garg, Gaur [129] which revealed an increased prevalence of anxiety among elderly individuals during the COVID-19 pandemic lockdown.

### Implications

The implications drawn from this study have significant relevance for health experts who frequently appear on TV talk shows and engage on social media platforms in Oman. In order to garner greater trust and credibility among the Omani population, these experts should take heed of the latest research findings regarding the interplay between COVID-19 anxiety and misinformation. Earlier studies, such as Price, Legrand [123], have suggested that individuals dealing with depression and previous post-traumatic stress disorder tend to turn to social media as a source of information during the pandemic to assuage their concerns. This aligns with the insights of Fabio and Suriano [130], who noted that digital media has assumed a pivotal role for information seekers during the COVID-19 pandemic. Notably, the advent of lockdown measures resulted in a remarkable 78% surge in digital media usage [130, 131].

The findings of this study unveil a substantial positive correlation between COVID-19 anxiety and the embrace of conspiracy theories (r = .225, p < .01), as well as the endorsement of misinformation beliefs (r = 163, p < .01). Given the prolonged duration of lockdowns and the continual evolution of COVID-19 and its variants, this discovery appears entirely rational. Prior research has also indicated that excessive use of digital media is closely linked to COVID-19 anxiety, implying that pandemic-related anxiety can detrimentally affect both working memory performance and information accuracy [130].

In light of these significant findings, it is strongly recommended that during pandemics, there should be concerted efforts by the public, civic, and private sectors to collaborate in providing psychological counseling and support, as well as financial assistance to individuals facing job loss. Furthermore, special attention should be given to extending social support to the elderly population, given their heightened vulnerability to potential complications stemming from pandemics [129]. Additionally, enhancing resilience in the face of adversity is suggested as a strategy to mitigate anxiety, thereby fostering positive long-term psychological and academic outcomes [132–134].

During pandemics, people are more likely to believe in conspiracy and misinformation beliefs especially when they use digital media [24]. As a result, policy makers should broadcast honest statements via traditional and digital media to debunk conspiracy theories and misinformation beliefs associated with health pandemics.

In conclusion, this study’s implications underscore the critical need for health experts and authorities in Oman to address COVID-19-related anxiety and misinformation through targeted interventions and support measures, emphasizing both practical and scientific directions. Policy recommendations should also be considered to guide the collective response to future pandemics.

### Limitations & future research

This study has several limitations that should be acknowledged. One limitation is the small number of participants from certain governorates, such as Alwusta, which presented challenges during the data analysis stage. Additionally, logistical challenges made reaching citizens from these areas difficult, highlighting the need for further research in these regions. Future studies should focus on these underrepresented regions to comprehensively understand the attitudes and beliefs surrounding COVID-19.

Another limitation of this study is that it did not explore the reciprocal relationships and influences between conspiracy theories, misinformation beliefs, and personality traits. Future research should investigate how these factors interact with one another in order to identify the underlying temperament and psychological factors that influence the inclination to advocate conspiracy theories or believe in certain misinformation. Additionally, given the high comorbidity between anxiety and depression [135], future studies should investigate the validity of depression in predicting conspiracy thinking and misinformation beliefs, particularly in the post-COVID-19 era, where recent statistics estimate an additional 76.2 million cases of anxiety worldwide due to the pandemic [122].

The timing of data collection also imposed limitations on this study. Data was collected after vaccines were widely disseminated and the pandemic was nearing its end. This may have contributed to the lack of significant effects observed in the SEM analysis. This was previously suggested by De Coninck, Frissen [24] who concluded that media effects would be more pronounced in regions where the pandemic reaches its peak.

Finally, the lack of previous research on conspiracy theories and misinformation beliefs in the Arab Gulf countries, specifically in Oman, represented a limitation in the background of this research. However, it also served as a motivating rationale for conducting this study. Future research should investigate the social and cultural factors underpinning COVID-19 conspiracy theories and misinformation beliefs and benefit from existing knowledge on conspiracy and misinformation to contain the spread of infodemics in emergency situations. Since many individuals are emotionally vulnerable to such critical circumstances [136], it is recommended to employ motivational and cognitive behavioral counseling in improving their vitality and mental health and reducing anxiety [137, 138].

## Study contribution

This study has contributed to our understanding of the importance of the veracity of information sources during health emergencies. The findings of this study emphasize that taking no action in building trust in these sources should not be an option. Additionally, the study provided insight into the association between three main challenges during the fight against the pandemic: conspiracy, misinformation, and anxiety. The effects of socio-demographic factors on the rampant misinformation and conspiratorial thoughts were also of significant importance in discovering their influences on these beliefs. This study highlights the need for continued research in this area to better understand the factors that contribute to the spread of misinformation and conspiracy theories during health crises and the impact they have on public health outcomes.

## Data Availability

Dataset is available on Mendeley: https://data.mendeley.com/datasets/tw6ms7w3mv/2.
